# Implementing exercise interventions in pediatric oncology: an expert consensus framework from the FORTEe project

**DOI:** 10.3389/fonc.2026.1893935

**Published:** 2026-07-08

**Authors:** Marie A. Neu, Elias Dreismickenbecker, Lena Wypyrsczyk, Filippo Spreafico, Adriana Balduzzi, Barbara Heißerer, Hayley Marriott, Norbert W. Paul, Eila Watson, Wilhelm Bloch, Martin Kaj Fridh, Miriam Götte, Lidija Kitanovski, Barbara Konda, Alejandro Lucia, Rodolf Mongondry, Katie Rizvi, Hanne Bækgaard Larsen, Nikolai Bauer, Ronja Beller, Carmen Fiuza-Luces, Gabriele Gauß, Mareike Kühn, Tommaso P. Moriggi, Olivia Pérol, Franziska Olivier, Domen Ravnik, Meta Rovan, Elena Santana-Sosa, Milica Stefanović, Peter Wright, William Zardo, Francesca Lanfranconi, Joachim Wiskemann, Joerg Faber

**Affiliations:** 1 University Medical Center of the Johannes Gutenberg-University Mainz, Childhood Cancer Center Mainz, Mainz, Germany; 2 Oncology Unit, IRCCS Istituto Giannina Gaslini, Genoa, Italy; 3 Pediatric Oncology Unit, Fondazione IRCCS Istituto nazionale dei Tumori, Milano, Italy; 4 Fondazione Monza e Brianza per Il Bambino e La Sua Mamma, Centro Maria Letizia Verga, Monza, Italy; 5 Pediatric Department, Fondazione IRCCS San Gerardo dei Tintori and School of Medicine and Surgery, Milano-Bicocca University, Monza, Italy; 6 Concentris research management gmbh, Fürstenfeldbruck, Germany; 7 Oxford Brookes University, School of Sport, Nutrition and Allied Health Professions, Oxford, United Kingdom; 8 University Medical Center of the Johannes Gutenberg-University Mainz, Institute for the History, Philosophy and Ethics of Medicine, Mainz, Germany; 9 Oxford Brookes University, Institute of Applied Health Research, Oxford, United Kingdom; 10 German Sport University Cologne, Department of Molecular and Cellular Sport Medicine at the Institute of Cardiovascular Research and Sports Medicine, Cologne, Germany; 11 University Hospital Copenhagen, Rigshospitalet, Department of Pediatrics and Adolescent Medicine, Copenhagen, Denmark; 12 University Hospital Essen, West German Cancer Center, Essen, Germany; 13 University Medical Center Ljubljana, Division of Pediatrics, Department of Haematooncology, Ljubljana, Slovenia; 14 Forma 3D Ltd., Forma 3D, Ljubljana, Slovenia; 15 Department of Sports Sciences, Faculty of Medicine, Health and Sports, Universidad Europea de Madrid, Madrid, Spain; 16 Centre de Lutte Contre le Cancer Léon Bérard, Prevention Cancer Environment Department, Lyon, France; 17 Youth Cancer Europe, Cluj-Napoca, Romania; 18 University Hospital Copenhagen, Rigshospitalet, Department of Clinical Medicine, Copenhagen, Denmark; 19 Heidelberg University Hospital and National Center for Tumor Diseases, a partnership between DKFZ and University Medical Center Heidelberg, Department of Medical Oncology, Working Group Exercise Oncology, Heidelberg, Germany; 20 Clinic of Pediatrics III, Hematology and Oncology, West German Cancer Center University Hospital Essen, Essen, Germany; 21 Hospital 12 de Octubre Research Institute, ‘imas12, Physical Activity and Health Laboratory, Madrid, Spain

**Keywords:** childhood cancer, exercise implementation, exercise intervention, pediatric oncology, physical activity, supportive care, training

## Abstract

**Background:**

Exercise therapy is increasingly recognized as an integral component of supportive care in pediatric oncology. However, exercise programs are not yet widely implemented nor established as standard care across Europe. The aim of this consensus statement is to provide practical recommendations for the implementation of exercise as a therapy in pediatric oncology, based on consensus from multi-disciplinary experts within the FORTEe project, funded by the European Union’s Horizon 2020 research and innovation programme.

**Methods:**

The FORTEe trial (NCT05289739) is a multicenter, randomized controlled trial aiming to investigate the effects of a structured exercise intervention in children and adolescents undergoing cancer treatment. Based on this collaborative experience, this expert-informed consensus statement, derived from a structured multinational trial preparation and implementation process, presents resource-adapted recommendations to support multidisciplinary teams in initiating, expanding and sustaining precision-based exercise programs in pediatric oncology.

**Results:**

The recommendations address key domains relevant to implementation, including human resources and interprofessional role distribution, staff training, medical clearance and reasons for adapting exercise, monitoring of exercise-related complications, practical delivery of exercise programs across diverse resource settings, as well as legal and regulatory requirements.

**Conclusion:**

These recommendations offer practical, context-specific guidance to support the stepwise implementation of exercise therapy in heterogeneous pediatric oncology settings. They are intended to facilitate the safe and sustainable integration of exercise as a therapy into routine childhood cancer care, regardless of local resource availability.

## 1 Introduction

In recent years, pediatric exercise oncology has evolved from a niche area of research to a rapidly expanding field, with programs emerging worldwide to support children and adolescents during and after cancer treatment (1–4). There are various benefits of physical activity and exercise interventions described in the literature and the described positive effects range from improved physical function and fitness (5, 6), reductions in common treatment-related symptoms such as fatigue (7, 8).

Taken together, the growing evidence of the multidimensional benefits of physical activity and exercise in children, adolescents, and young adults (CAYA) with cancer highlights its clinical relevance within supportive care (3, 5, 8–12). In response to these effects, a growing number of structured exercise and physical activity programs have been developed and implemented in various clinical settings. Many approaches offer multimodal exercise interventions that combine different motor capacities like endurance, strength, flexibility, and coordination training (5, 7, 13, 14). While some are implemented on an outpatient and/or inpatient basis within hospitals (5, 13, 14), others include home-based training either as a supplement or as a standalone option (7, 15, 16). These home-based interventions often incorporate digital tools such as video-based exercise sessions (17). The level of professional supervision of exercise programs varies across settings, ranging from fully supervised interventions to partially or fully unsupervised approaches, for both in-hospital and home-based interventions. These formats are often combined in hybrid models, such as integrating supervised in-hospital sessions with structured home-based training supported by regular remote follow-ups (18–20). However, supervised training programs have been shown to offer advantages over unsupervised approaches (21).

Despite a growing body of evidence describing a broad range of exercise intervention approaches and program models in pediatric oncology, translation into routine care remains limited. Access to structured exercise interventions in pediatric oncology remains inconsistent worldwide. While disparities are often more pronounced in resource-constrained settings, limited availability is not restricted to low- and middle-income countries but also reflects differences in infrastructure, expertise, and institutional priorities across high-income settings.

Exercise interventions have been shown to be feasible and safe (22–24). Moreover, various studies showed beneficial effects on physical and psychosocial outcomes (5, 7, 8, 25), and early adoption has occurred in a number of clinical settings (26). However, exercise programs are not yet widely implemented nor established as standard care across Europe (2). This discrepancy between existing knowledge, recommendations in pediatric exercise oncology guidelines and practice highlights the need for clear, practical frameworks and pragmatic guidance to support multidisciplinary teams in initiating, scaling, and sustaining exercise programs across diverse healthcare settings (8). In line with this, previous studies performing a comprehensive needs assessment demonstrated strong professional support for exercise as a form of supportive therapy, while simultaneously highlighting substantial gaps in research translation and implementation structures (2, 27).

Exercise oncology in adults is supported by well−established guidelines, for example, the Exercise, Diet, and Weight Management During Cancer Treatment: American Society of Clinical Oncology Guideline (28) or American College of Sports Medicine roundtable on exercise guidelines for cancer survivors (29). For adults, evidence shows that exercise is safe and improves physical function, quality of life, and reduces fatigue in several cancer survivor groups (28, 29). Exercise has even been shown to improve survival (30).

While adult exercise oncology is well established, research and guidelines for children are still emerging. Within the International Society of Paediatric Oncology (SIOP), experts from around the world convene and, through its Rehabilitation and Physical Medicine Special Interest Group, advance guideline development and research prioritization for pediatric oncology rehabilitation. The International Pediatric Oncology Exercise Guidelines (iPOEG) (3) emphasize that exercise is both possible and important for all children and adolescents with cancer, regardless of age, ability, diagnosis, treatment phase, or setting (e.g., hospital, community, school, or nursery). According to the guidelines, daily activity levels may vary depending on the child’s condition. The iPOEG also, recommend, close communication between exercise and healthcare teams to ensure patient safety, particularly in uncertain cases. The exercise program should be prescribed by qualified professionals with pediatric oncology expertise, considering individual factors such as age, diagnosis, treatment, and personal preferences (3).

Recent national guidelines for Germany, provided by the multidisciplinary Network ActiveOncoKids (NAOK) (31), further emphasize that exercise programs for children and adolescents with cancer should be individualized, consider safety aspects, and integrated into clinical care. Hospitals should provide exercise-friendly settings with accessible spaces and qualified staff. Exercise sessions should be tailored to the preferences, health status, and motivation of CAYA with cancer, including the choice of activities, frequency, intensity, and duration. While there is now consensus that supervised exercise interventions should be integrated throughout treatment and survivorship, delivered by trained professionals who can adapt the program to individual clinical and functional limitations. Recent studies have begun to explore how pediatric exercise programs can be effectively implemented (26, 32–34), but additional research is still needed to establish practical frameworks for widespread application. Furthermore, the performance of exercise sessions should be supported by structured communication and interdisciplinary collaboration to overcome barriers, promote adherence, and enhance quality of life (31).

## 2 Methods and aims

### 2.1 Aims

Despite increasing knowledge and the availability of international and national recommendations, substantial variability remains in how exercise programs are implemented in pediatric oncology care. We therefore aim to bridge the discrepancy between research and practice by consolidating expert consensus, emphasizing a tiered, resource-stratified implementation approach, detailing scalable tools and strategies suited to different levels of infrastructure and personnel, to support multidisciplinary teams in initiating, expanding and sustaining exercise programs in pediatric oncology.

The consensus statement is targeted at pediatric oncologists, nurses, exercise professionals and associated healthcare providers as well as to patients, parents and survivor associations, and aims to deliver practical guidance on implementation of exercise programs, considering different resource levels.

#### 2.1.1 Overview of the FORTEe trial and consortium

The FORTEe trial (35) (ClinicalTrials.gov identifier: NCT05289739) is a multicenter, randomized, controlled, Horizon 2020-funded trial evaluating the effects of a structured exercise intervention in children and adolescents undergoing cancer treatment. Participants are enrolled across ten sites in seven European countries (see **Table 1**) and the trial integrates inpatient, outpatient and home-based components. The FORTEe consortium draws on a collaborative network of exercise professionals, pediatric oncologists, nurses, physiotherapists, social workers, and other healthcare professionals. In this manuscript, the term exercise professionals refers to individuals with formal training in exercise science, physiotherapy, clinical exercise physiology, or related disciplines, with additional expertise in pediatric oncology where available. In addition, patient and family perspectives were represented throughout the FORTEe project through the active involvement of Youth Cancer Europe, survivor advocacy groups, and parent associations across participating countries (e.g., Fondazione Maria Letizia Verga, Monza, Italy). Youth Cancer Europe, a consortium partner organization represented by co-author Katie Rizvi, contributed to trial design discussions, implementation planning, consortium meetings, and manuscript development, thereby ensuring that patient and survivor perspectives informed the consensus process. Furthermore, perspectives from consortium members, including exercise professionals with personal lived experience of cancer, contributed to the design and delivery of the intervention as well as to the development of the present recommendations. These patient, survivor, and family perspectives contributed to discussions regarding flexibility of participation, age-appropriate exercise delivery, home-based training opportunities, family involvement, and long-term accessibility of exercise programs, and are reflected in the resulting recommendations. In addition, lived experience perspectives contributed to the training and mentoring of newly involved healthcare and exercise professionals, supporting the development of patient-centered implementation approaches across participating sites.

**Table 1:** Overview of the institutions participating in the FORTEe trial.

FORTEe trial sites	Country
Universitätsmedizin der Johannes Gutenberg-Universität Mainz (UMC-Mainz)	Germany
Universitätsklinikum Heidelberg (UKHD)	Germany
Centre de Lutte Contre le Cancer Leon Berard (CLB)	France
Oxford Brookes University (OBU) in cooperation with the Oxford University Hospital Trust (John Radcliff Hospital and Churchill Hospital)	United Kingdom
Fondazione Monza e Brianza per il Bambino e la sua Mamma (MBBM)	Italy
Region Hovedstaden (RegionH)	Denmark
Universidad Europea de Madrid SL (UEM) in cooperation with the Hospital Infantil Universitario Niño Jesús and the Hospital Universitario 12 de Octubre	Spain
Fondazione IRCCS Istituto Nazionale dei Tumori (INT)	Italy
Univerzitetni Klinični Center Ljubljana (UKCL) in Cooperation with Forma 3D Ltd. (Forma3D)	Slovenia
Universitätsklinikum Essen (UKESSEN)	Germany

Based on the current state of literature and the recent experiences during the implementation and performance of the FORTEe trial regarding critical enablers and barriers at varying levels of resource availability, staff expertise, and organizational readiness, the FORTEe Consortium has developed this expert consensus statement to provide recommendations for implementing exercise as a therapy in pediatric oncology that are stratified by resource availability and setting. The FORTEe expert consortium comprised 35 members, encompassing 23 exercise professionals (including sports medicine physicians, exercise scientists and kinesiologists), six pediatric oncologists, one specialist in supportive cancer care, one public management expert, one health services or administrative scientist, one social and nursing scientist, one medical ethicist and one patient advocacy representative (Youth Cancer Europe), alongside several contributors with lived experience of childhood cancer.

#### 2.1.2 Implementation context within the FORTEe trial

Within FORTEe, the resource level/setting prior to the implementation varied substantially across participating trial sites. During the preparation phase, a systematic cross-site comparison revealed notable heterogeneity with regard to staffing structures, available exercise space, exercise equipment, and the institutional integration of exercise services. To reflect this heterogeneity, resource-stratified implementation settings for exercise interventions in pediatric oncology are summarized in **Table 2**. Through iterative discussions during Clinical Partner meetings and Steering Committee meetings, these differences were consolidated into three pragmatic resource levels/settings (basic, intermediate and enhanced) across defined domains (staff, exercise space and equipment). The resulting three-level framework was further refined during the early implementation and monitoring phases to reflect operational realities and scalability considerations.

**Table 2:** Resource Stratified levels/settings for the Implementation of Exercise Interventions in Pediatric Oncology.

	Resource level / setting**		
Resource domain	Basic	Intermediate	Enhanced
Staff	No staff formally trained in exercise delivery within pediatric oncology.	At least one trained exercise professional or other trained staff member, at least available on a part time basis.	Multi-professional team including exercise professionals integrated into pediatric oncology care and with regular availability.
Exercise Space*	No dedicated or regularly accessible space suitable for exercise activities.	Small or shared space suitable for exercise activities, with limited or inconsistent accessibility.	Dedicated exercise facilities with regular and reliable accessibility and visibility.
Equipment*	No professional exercise equipment available.	Basic exercise equipment available, not covering all training modalities optimally.	Comprehensive professional equipment available, covering strength, endurance, coordination and playful exercise formats.

*Resource specific recommendations are described below and are included in detail in **Supplementary Material 3**.

**While structural resources (staff, space and equipment) across basic, intermediate and enhanced levels were stratified, a set of core safety requirements remains essential across all settings. These include prior medical clearance, continuous clinical communication between medical and exercise professionals, individualized exercise prescription and adaptation based on current clinical status, and systematic monitoring and documentation of exercise-related symptoms or adverse events. These safety principles are required for safe implementation and apply independently of resource level.

This table describes resource-based implementation levels/setting across three domains: staff, exercise space, and equipment. Domains are presented separately, as individual centers may demonstrate different resource levels across domains, with potential overlap between levels. The terms basic, intermediate, and enhanced indicate increasing availability of resources for exercise implementation and do not imply differences in the quality of oncological care.

Resource levels may overlap, and individual centers may demonstrate different levels across resource domains. The levels represent gradations of implementation capacity and do not imply differences in the quality of oncological care delivered at participating centers.

Among the FORTEe trial sites, some hospitals already had an established exercise program, including dedicated personnel and fully equipped training facilities within the hospital, allowing direct and immediate access to CAYA with cancer. Other sites, however, lacked trained in-house staff for exercise programs. In these settings, adaptive implementation solutions were often required, such as the involvement of external exercise professionals, as illustrated in the case study below.

To address this substantial heterogeneity in local resources and to ensure feasible and standardized implementation across all participating sites, the FORTEe trial provided the necessary structural and organizational framework. Where necessary, FORTEe facilitated the employment of exercise professionals, supported the establishment of initial exercise therapy structures, and provided study-specific training for the delivery of exercise interventions and fitness assessments. Additionally, the coordination team provided standardized materials for exercise testing and training sessions, as well as video-based exercise resources to support program implementation. By creating these resources and structures, FORTEe established a potential foundation for subsequent integration of exercise into routine pediatric oncology care at participating sites, particularly where such services had not previously existed.

These measures represent the core components of the FORTEe implementation approach, which will be illustrated in the following case study from the Ljubljana site, before presenting the concrete FORTEe recommendations derived from this approach.

Several components of the FORTEe implementation approach were specific to the context of a funded multicenter clinical trial. These included project-supported coordination, site preparation, staff training, standardized study materials, trial-specific safety and monitoring procedures, and digital tools developed within FORTEe to support and enhance the exercise intervention. Similarly, the FORTEe Exercise Training and Testing Booklets were developed within the project context, although they have since been published and disseminated to support broader implementation beyond the trial. These FORTEe-specific components should therefore be understood as illustrative implementation resources rather than mandatory prerequisites for routine clinical practice.

In contrast, the transferable elements of the framework are the underlying implementation principles: structured medical clearance, individualized and supervised exercise prescription, predefined criteria for adapting or temporarily pausing exercise, continuous communication between clinical and exercise teams, systematic monitoring and documentation of exercise-related events, and resource-stratified planning of staff, space, and equipment. The FORTEe exercise intervention was deliberately designed to be deliverable across heterogeneous inpatient, outpatient, and home-based settings and was implemented across trial sites with differing resources and healthcare contexts. Accordingly, the framework is intended to support local adaptation of pediatric exercise oncology services while preserving core safety and implementation principles.

#### 2.1.3 Development of the consensus framework

The recommendations presented in this manuscript emerged from a structured, governance-based consensus process that was embedded within the one-year preparation phase and the subsequent initiation of the multi-center FORTEe trial. During this period, the Steering Committee, General Assembly and the Clinical Partners held regular meetings. As part of these meetings, different professional groups of the multidisciplinary FORTEe consortium contributed complementary perspectives throughout the process, including medical safety and clinical feasibility (pediatric oncologists), exercise prescription and program adaptation (exercise professionals), workflow integration, patient motivation and participation barriers (nurses and other clinical staff), ethical and patient-centered considerations (social scientists and ethicists), as well as digital implementation approaches (technology developers and digital health experts). All meetings were formally documented and the minutes were circulated among consortium members and approved.

The Clinical Trial Management Committee developed core documents, including medical clearance criteria, exercise adaptation guidance, intervention manuals and safety monitoring procedures. Drafts were version-controlled and circulated for written feedback across participating sites. Proposed revisions, including critical objections and alternative formulations, were discussed in follow-up meetings or via written feedback-rounds. Where differing opinions or unresolved points emerged, recommendations were revised and further discussed iteratively until documented pragmatic agreement regarding feasibility, safety and applicability across participating sites was achieved. Final recommendations were incorporated into the complete implementation framework only after these unresolved points had been addressed and formal approval had been reached within the established consortium governance structure. Overall, this approach represents an interdisciplinary, governance-based consensus process embedded within the multicenter trial implementation process, characterized by repeated interdisciplinary discussion, iterative revision cycles, transparent documentation, moderated resolution of objections and final approval processes.

The recommendations were further refined during early implementation and cross-site monitoring and subsequently consolidated during manuscript preparation through additional interdisciplinary review. Thus, the resulting framework represents a multinational, multidisciplinary expert consensus based on prospective trial preparation, shared implementation experience and established pediatric exercise oncology guidelines.

## 3 Case study: implementation of exercise therapy within pediatric oncology in Slovenia

This case study, based on our shared experiences, provides practical examples of the challenges and opportunities of implementing exercise interventions in pediatric oncology. Slovenia has a highly centralized pediatric oncology care structure, with all children and adolescents with cancer being treated at a single national center (36), the University Medical Center Ljubljana, with an annual incidence of up to 85 pediatric cancer cases (37). Prior to participation in the FORTEe trial, no structured exercise therapy program existed within pediatric oncology care in Slovenia, and exercise professionals were not integrated into hospital-based oncology teams. This context provided a unique opportunity to implement exercise therapy from the ground up within a nationally centralized healthcare system.

Implementation at the Ljubljana FORTEe site was conducted through a close collaboration between the clinical team at the University Medical Center Ljubljana and the consortium partner Forma 3D, a small- and medium-sized enterprise with expertise in physical activity promotion in children and adolescents, exercise-based interventions, and youth-focused health and education programs, under guidance of the FORTEe coordinating center and relevant work package leads. Initial key barriers included the absence of a dedicated exercise space, limited institutional familiarity with exercise based supportive care and regulatory requirements related to professional roles and participant safety. Exercise sessions were therefore initially delivered in non-dedicated spaces such as patient rooms and available ward areas, using low cost and adaptable equipment. Two kinesiologists were recruited, following approval through the national health authority. They received structured FORTEe specific training, including core competencies related to medical clearance, exercise adaptation, and patient safety (described in detail below), and parallel education was provided to oncologists and nursing staff to ensure appropriate clearance procedures and safe integration into daily clinical routines. Further, staff training comprised structured on-site placements of the Ljubljana team at more experienced FORTEe consortium centers, combining observational exposure with supervised practical training, alongside on-site field visits to Ljubljana conducted by representatives of the FORTEe coordinating center and clinical trial management team.

Through sustained interprofessional collaboration and institutional support, the kinesiologists became formally recognized as healthcare professionals within the hospital and integrated into the pediatric oncology care team.

During the FORTEe trial period, the Ljubljana site enrolled 42 participants between December 2023 and June 2024, exceeding its initial recruitment target of 30 participants. In addition, 511 supervised exercise sessions were delivered as part of the intervention. These implementation metrics provide initial objective indicators of program uptake and operational feasibility within a setting where no structured pediatric oncology exercise program had previously existed.

Importantly, these metrics should be interpreted within the Slovenian clinical and regulatory context. At the start of FORTEe, structured supervised exercise therapy had not yet been integrated into routine pediatric oncology care at University Medical Centre Ljubljana, and kinesiologists had not yet been formally embedded within the Slovenian healthcare workforce framework, limiting their systematic appointment to clinical positions. FORTEe-related implementation experience and engagement with institutional and national stakeholders helped support recognition of this workforce need and the subsequent inclusion of the profession under the official title “kinesiologist in healthcare” among healthcare associates/allied health professionals in the Slovenian Ordinance on the List of Professions for Healthcare Activity. This regulatory development facilitated the establishment of kinesiologist positions at University Medical Centre Ljubljana and supported continuation of exercise-based supportive care beyond the trial period.

Building on these developments, exercise therapy was progressively embedded into routine ward processes and a sustainable structure was established through the employment of a full-time kinesiologist. In addition, collaboration with the parents’ association “Junaki 3. Nadstropja” enabled the preparation, approval, and financing of a dedicated exercise room, securing long-term access to exercise therapy beyond the trial period. This case illustrates how a clinical trial can act as one potential catalyst for system-level change, demonstrating the feasibility of stepwise implementation of exercise therapy in pediatric oncology, even in settings without pre-existing infrastructure.

## 4 FORTEe recommendations

Based on the resource stratification framework described above (**Table 2**), recommendations for implementation were developed within FORTEe to support stepwise establishment and scaling of exercise interventions in pediatric oncology. Guidance focuses on pragmatic actions that centers can take within existing constraints and is intended to facilitate progression within and across resource domains. The guidance addresses structural and organizational aspect critical to a successful implementation. A summary of the core recommendations for implementing exercise in pediatric oncology, derived from the FORTEe consortium experience, is provided in **Table 3**.

**Table 3:** Core Recommendations for Implementing Exercise Therapy in Pediatric Oncology, derived from the FORTEe consortium experience.

Implementation domain	Core recommendation
Human Resources and Team Structure	• Define clear roles for an interdisciplinary team (physicians, exercise professionals, nurses and psychosocial staff). • Ensure medical oversight and adapt staffing to local resource availability. • Establish regular clinical exchange to anchor exercise therapy within the clinical structure. • Ensure a continuous flow of real-time clinical updates to adapt training safely. • Provide structured education for exercise professionals (e.g., medical clearance, safety criteria, exercise testing) • Promote awareness among physicians and nurses to foster clinical endorsement.
Medical Clearance and Adaptation Criteria	• Implement standardized joint medical clearance procedures.
Monitoring for Exercise‐Related Complications	• Establish structured documentation of exercise-related events using standardized templates; ensure prompt interdisciplinary communication.
Performing exercise programs in pediatric oncology	• Deliver individualized, supervized, voluntary, age-adapted and treatment-phase-specific exercise sessions across inpatient, outpatient and home-based settings when feasible. • Provide safe, visible and accessible exercise areas, ranging from shared ward spaces to dedicated facilities/gyms. Resource-stratified operational guidance is provided in **Supplementary Material 3**. • Use disinfectable, age-appropriate equipment, ranging from simple portable materials to comprehensive professional infrastructure. Resource-stratified examples are provided in **Supplementary Material 3**.
Legal and Regulatory Considerations	• Clarify scope of practice, liability/insurance (including professional indemnity) and institutional approval requirements prior to program initiation.
Technologies and Digital Resources	• Integrate digital tools (apps, telehealth) as complementary, motivational, and low-threshold tools, particularly in isolation, for home-based training, or when staff is limited.

This table summarizes the main structural and organizational elements for implementing exercise programs in pediatric oncology care. The recommendations are derived from the multinational FORTE consortium’s experience with trial preparation and early implementation, and reflect a structured expert consensus process. The recommendations are intended to support adaptation across heterogeneous healthcare environments. Detailed, resource-stratified operational recommendations regarding exercise space and equipment are provided in **Supplementary Material 3**.

### 4.1 Recommendations regarding Human resources, interprofessional team roles and responsibilities, and staff training

Implementing exercise programs in pediatric oncology requires a coordinated, interdisciplinary structure in which physicians, exercise professionals, nurses and psychosocial professionals jointly integrate physical activity and exercise into routine care (3, 38, 39). Although clinical structures, especially regarding the provision of exercise programs, may differ across childhood cancer centers in Europe (3), including those involved in FORTEe, the following framework proved practicable in the setting of the FORTEe trial. It represents an adaptable model rather than a fixed scheme.

Clinical staff (physicians and nurses) should bear primary responsibility for granting medical approval for enrolment in exercise programs, ensuring that all interventions are implemented within a framework of clinical safety and individual appropriateness (3). Their explicit endorsement of physical activity during routine consultations may increase engagement of CAYA with cancer, emphasizing exercise as a medically meaningful component of cancer care (39). To support consistent decision-making, the FORTEe consortium has defined criteria for medical clearance and exercise adaption (see below) (35). This shared guidance has enabled centers to adapt recommendations to their local context while maintaining common safety standards.

Exercise professionals should design and deliver personalized exercise programs tailored to the child’s clinical, physical, and psychosocial context (3). To enable this individualization and ensure that every activity is both safe and purposeful, program adaptations should be based on two core information streams (1): real-time clinical updates (e.g., blood counts, acute symptoms, cancer treatment); and (2) standardized performance assessments that track functional changes (3, 29). Exercise professionals should flexibly and safely adapt exercise content to different age groups, diagnoses, and treatment phases, as well as to diverse clinical settings, aligning with clinical circumstances and the needs of CAYA with cancer to maintain continuity and accessibility (3, 40). In addition, exercise professionals may provide ongoing counselling, develop progressive training plans, and support the transition to survivorship by connecting families with aftercare programs, community sports clubs, or home-based physical activity resources.

Psychosocial professionals (clinical psychologists, social workers, and related staff) may play a key role in addressing motivational, emotional, and family-related factors that influence participation. They may assist recruitment, identify barriers such as anxiety, fatigue or family stress, and collaborate closely with exercise therapists to integrate psychosocial needs into session planning.

Across all phases, interdisciplinary communication, through regular clinical exchange, case conferences and tumor boards, should ensure coherent decision-making and anchor exercise therapy as an integral, patient-centered part of childhood cancer care (3, 39). This communication structure ensures a closed-loop process linking medical clearance, real-time monitoring, and adaptive decision-making throughout the exercise intervention.

Structured and ongoing education for exercise professionals, physicians, nurses, and allied health professionals is considered essential to ensure appropriately tailored exercise prescriptions, enhance clinical awareness, and support consistent, evidence-based safety decisions (3, 41, 42).

Accordingly, within the FORTEe trial, a standardized staff training program tailored to the different professions was implemented, covering medical clearance procedures and clinical criteria requiring exercise modification, exercise testing, and the personalization and delivery of exercise sessions for children and adolescents undergoing cancer treatment to ensure safe and consistent exercise therapy across sites.

With the growing role of exercise oncology in pediatric cancer care and the development of corresponding guidelines (3, 43), the first structured education programs are beginning to emerge. An example is the German Network ActiveOncoKids (NAOK) “Exercise Interventions in Pediatric Oncology” training (44), which qualifies exercise professionals to deliver exercise therapy to CAYA with cancer. Additional educational initiatives for medical staff are needed to promote awareness, foster interdisciplinary collaboration, and facilitate the clinical implementation of exercise programs in pediatric oncology. Although currently applied on a trial specific basis within FORTEe, such educational strategies represent a transferable prerequisite for the sustainable integration of exercise into routine clinical practice across Europe.

According to the resource stratification framework, centers operating in basic resource settings should prioritize qualifying at least one staff member in pediatric exercise oncology, supported by standardized training and clear medical clearance pathways. This enables safe and feasible exercise delivery, even without dedicated infrastructure. In intermediate settings, the focus is on expanding staff capacity by ensuring the regular availability of trained exercise professionals, strengthening interdisciplinary collaboration, and embedding exercise therapy more consistently into clinical workflows. This facilitates progression toward integrated, routine exercise care.

### 4.2 Recommendations on medical clearance and adaptation criteria

Medical clearance before enrolment to an exercise program is a fundamental component of safe practice in pediatric oncology, as children undergoing cancer treatment frequently experience fluctuating clinical conditions, acute toxicities, and treatment-related complications that can influence exercise tolerance and risk. Evidence from exercise-oncology guidelines highlight the need for structured screening to identify contraindications, guide adaptations, and support safe exercise delivery across all phases of treatment (3, 29). Several childhood cancer centers and guideline initiatives have already specified safety and adaptation criteria (3, 43, 45), for example within the international Pediatric Oncology Exercise Guidelines (iPOEG) (3), demonstrating the growing relevance of standardized pre-exercise assessment in clinical practice.

Building on existing literature, the FORTEe consortium developed study-specific medical-clearance criteria informed by both a review of the existing literature and expert consensus across participating trial sites. Although designed for the FORTEe clinical trial, these criteria reflect widely applicable principles and may serve as a transferable framework for medical decision-making in pediatric exercise oncology.

The FORTEe recommendations outline indications for exercise clearance before both exercise testing and training, as well as criteria for modifying or temporarily withholding exercise in response to clinical findings. Medical clearance should ideally be performed jointly by the treating physician and the exercise professional and is tailored to each child prior to every exercise session. Exercise professionals receive ongoing clinical updates (e.g., laboratory values, treatment status, acute symptoms) and should be integrated into ward-based workflows such as clinical rounds, interdisciplinary meetings, or case discussions, depending on local site structures. During exercise sessions, clinical tolerance should be continuously monitored, including observation of fatigue or distress and use of perceived exertion scales (e.g., Borg scale); additional physiological monitoring may be used when clinically indicated and locally available.

In clinical practice, medical clearance is therefore complemented by continuous interdisciplinary communication and real-time monitoring during exercise sessions. Based on this combined information flow, exercise professionals and physicians jointly decide whether exercise can be continued, adapted, or temporarily paused.

Importantly, these safety requirements apply across all resource levels and should be maintained even in basic resource settings, where limitations in staffing, space, or equipment must not compromise medical clearance, clinical communication, individualized adaptation, or monitoring procedures.

Detailed criteria are published separately (35), core criteria are provided in **Supplementary Material 1**.

### 4.3 Recommendations on monitoring for exercise‐related complications

Systematic monitoring is essential to ensure safety during exercise interventions in CAYA with cancer. A retrospective nationwide German study (22) including 35,110 supervised sessions reported a very low rate of adverse events, with only six Grade 2 to 3 events (0.017%) and no life-threatening events or serious adverse events during acute treatment. Minor events occurred in 2.8% of sessions, most commonly muscle soreness and transient circulatory or abdominal symptoms (22). The findings suggest that supervised exercise is safe in this population, with severe adverse events being rare during exercise sessions. Nonetheless, ongoing vigilance for exercise‐related events remains essential across all tiers.

Therefore, in the FORTEe trial, exercise-related safety was further supported by a standardized system for documenting serious exercise-related complications (SERCs) (35). Standardized incident‐reporting templates were developed and used within FORTEe to consistently document date/onset, event type, grade, case description, relationship to exercise as well as actions taken (see **Supplementary Material 2**). The SERC documentation was designed to capture a broad range of potential, exercise-related complications across multiple organ systems.

Outside the context of clinical trials and when implementing clinical exercise oncology, exercise professionals should also document any adverse signs or symptoms, communicate them promptly to the medical team, and where possible, report them to a register (46). Standardized incident‐reporting templates can help to ensure consistent capture of exercise related complications and their detailed characteristics. This might contribute to continued safety and quality assurance in clinical exercise programs.

### 4.4 Recommendations on performing exercise programs in pediatric oncology

In pediatric oncology, exercise programs must balance safety, feasibility, and individualization while accounting for patient heterogeneity with respect to age, diagnoses, treatments, and clinical conditions. Participation in exercise sessions should be voluntary, and CAYA with cancer should be encouraged to actively express their preferences, including the option to decline participation on a given day and re-engage at a later time point. This approach acknowledges fluctuating physical and emotional states during treatment and supports the individual’s autonomy.

Due to the significant differences in infrastructure and requirements among the participating European FORTEe trial sites, the program was designed to be implementable across all countries and centers, despite varying local conditions. This design aimed to ensure consistent delivery of the sessions, regardless of available rooms or equipment.

In alignment with existing guidelines on exercise therapy in pediatric oncology (3, 31), exercise should be offered as frequently as possible, ideally on a daily basis, where feasible and clinically appropriate. Consistent with previous exercise intervention studies, the FORTEe exercise intervention (35) aimed to deliver three to five sessions per week, each lasting 45 to 60 minutes. The exercise program consisted of a combination of endurance, strength, flexibility, coordination/balance, and gait training. To maintain motivation and adherence, particularly among younger children, sessions were designed to be playful. The content, duration, and intensity of each session were adjusted according to the patient’s functional capacity, age, fitness level, overall health status, and personal preferences (3, 40).

During outpatient periods and weekends while hospitalized, for example, participants had the opportunity to train independently based on recommendations from the exercise professionals.

To provide specific, actionable recommendations that support the implementation of exercise programs in pediatric oncology, the FORTEe Consortium additionally developed its Exercise Training and Testing Booklets (47). They include exercises targeting cardiorespiratory fitness, strength, balance, and flexibility, which can be performed with minimal equipment or simple tools such as resistance bands or dumbbells, facilitating integration into routine clinical care. Each exercise is presented in multiple difficulty levels to accommodate varying physical conditions and treatment phases. The booklets also incorporate didactic physical activity games with therapeutic play scenarios to enhance motivation and enjoyment. Furthermore, they provide targeted recommendations for managing specific clinical conditions, including respiratory, cardiac, musculoskeletal, and neuromuscular impairments.

Depending on the resources of each trial site, they could also use digital tools (see recommendation on Technologies and Digital Resources) for support. Supervised home-based training was also possible via a video platform. To maintain motivation during home-based training, efforts were made to include parents, siblings, and other caregivers in the sessions whenever possible. This family-centered approach reflects feedback received from patient and parent representatives during FORTEe program development, who highlighted the importance of integrating exercise into daily family routines whenever feasible.

Although specific materials, manuals and digital tools were developed within the FORTEe trial, these are presented as illustrative examples of scalable implementation tools rather than prescriptive components. The transferable elements lie in the underlying principles: structured medical clearance, individualized exercise prescription, interdisciplinary collaboration, safety governance and adaptable delivery formats across varying resource levels. The present manuscript therefore focuses on organizational and structural enablers rather than on the specific exercise protocol itself.

Centers seeking to establish exercise services for childhood cancer patients may use the FORTEe approach and adapt it to local conditions. In the FORTEe program, sessions were primarily led by specialized exercise professionals who were available almost every day at most sites. However, in centers with basic staff level (no staff formally trained in exercise delivery within pediatric oncology), sessions may, where appropriate, initially be delivered by other appropriately trained personnel (e.g., nurses, or rehabilitation staff) depending on local structures and competencies. Therefore, a stepwise implementation is reasonable.

To *de-novo* implement an exercise program, comprehensive sports infrastructure is beneficial but not mandatory. Depending on the patients’ clinical status and the available space, centers operating in basic resource settings (**Table 2**) may deliver exercise sessions in patients’ rooms or in other suitable open hospital areas, such as ward corridors or multipurpose rooms, preferably located in easily accessible and visible areas to increase patient adherence and facilitate prescriber oversight, allowing flexible delivery without requiring dedicated facilities (3). Centers with intermediate resource settings should seek semi-dedicated spaces that are regularly accessible, easily reachable, and visible, even if they are shared with other activities. This will facilitate more consistent scheduling and progressive training. Similarly, the use of specialized exercise equipment is helpful, but not mandatory. In basic resource settings, exercise sessions can be implemented using body weight alone or simple household items, such as water bottles, towels, or everyday materials used for therapy. Intermediate settings may provide basic exercise equipment covering key training modalities, to allow more tailored and progressive exercise prescriptions. Overall, stepwise implementation is recommended, starting with available resources and gradually expanding staff capacity, exercise space, and equipment as feasible. Designing clinical spaces with low barriers, good visibility, and easy accessibility that facilitate exercise and encourage spontaneous activity is helpful for integrating exercise into daily ward routines.

In order to prevent infection, all materials used during sessions must be easy to disinfect, and cleaning procedures must adhere strictly to local clinical standards.

Overall, experience from FORTEe suggests that exercise programs for childhood cancer patients undergoing intensive treatment can be implemented, even under (temporarily) limited structural and staffing conditions; however, such conditions should remain short-term, and it is highly recommended that programs are led by professionals specialized and trained in pediatric oncology.

Ideally, the approach is flexible, build on existing resources, and allow for stepwise expansion (see **Table 1**). Further resource specific recommendations are included in **Supplementary Material 3**.

### 4.5 Recommendations on ethical, legal, regulatory, and institutional requirements

Ethically sound pediatric research must address real-world burdens and emotional dynamics beyond procedural compliance. Findings from the FORTEe trial staff survey highlight the importance of flexible, child-centred approaches, sustainable access to beneficial interventions, and institutional structures that promote ethical reflection (48). In addition, ethical implementation frameworks should consider equity in access to exercise interventions across heterogeneous healthcare environments. Resource-adapted delivery models may help reduce disparities between centers with differing levels of infrastructure, staffing, and supportive care resources, thereby supporting broader access to exercise-based supportive care in pediatric oncology.

The legal requirements for conducting supervised exercise sessions vary substantially by institution, region, and national legislation. Although FORTEe participants were covered by a trial-specific insurance policy, centers planning to implement a new exercise program should review local regulatory, labor, and liability requirements before doing so.

This includes clarifying the locally applicable scope of practice and qualification framework for delivering exercise sessions in hospitals, which may differ by country. The local/national accreditation, registration and continuing education requirements relevant to exercise staff should also be clarified.

Additionally, centers should review insurance policies and reimbursement frameworks to understand coverage of supportive care services, including exercise therapy or physiotherapy. They should also determine if pre-authorization procedures, or regional reimbursement policies apply. Liability and indemnity arrangements should also be clarified, including professional liability insurance for exercise staff and confirmation that institutional or employer based policies cover supervised exercise interventions and potential adverse events. Clinical governance approval for designated exercise spaces as well as compliance with institutional fire safety, infection control, and occupational health regulations should be ensured. Engaging with relevant hospital departments early on, including legal services, risk management, and facilities management, can facilitate the timely adaptation of sites and support the sustainable integration of exercise programs into pediatric oncology care.

### 4.6 Recommendations on technologies and digital resources

Technologies can be useful for encouraging exercise in children with cancer, particularly when staffing resources are limited, patients are at home or during periods of medical isolation. Emerging evidence in pediatric oncology suggests that technology-based interventions are feasible and may improve physical and psychosocial outcomes (18, 49).

Within the FORTEe project, several technologies were developed to support exercise training in pediatric oncology. These digital tools include the “FORTEe Get Strong” app, a gamified mobile app, that promotes physical activity and provides information about health-related behaviors (50). Another app uses augmented reality (AR) to allow users to perform strength-based exercise sessions guided by an animated avatar in an AR environment (51, 52). In addition to these two apps, there is a motion-tracking system (Pixformance) where an avatar demonstrates exercises while real-time feedback on exercise execution is provided. Furthermore, the FORTEe trial included telehealth-supervised exercise sessions to support participants during home training. These digital tools were developed and applied within the FORTEe trial primarily as implementation-support instruments to facilitate delivery, engagement, and accessibility of the exercise intervention, particularly in home-based or resource-limited settings.

Although the independent effectiveness was not evaluated within the scope of this consensus statement, experience from FORTEe illustrates how digital solutions can support exercise programs and enhance flexibility, engagement, and accessibility (50, 51). Centers seeking to implement pediatric exercise oncology programs may benefit from integrating technology as a complementary, motivational and low-threshold tool, particularly in settings with limited staff or when distance-based support is necessary.

## 5 Limitations and transferability considerations

Several limitations should be considered when applying this framework beyond the FORTEe trial context. First, the framework was developed within a funded multicenter research setting that provided structured support for staffing, training, coordination, and standardized materials, which may not be readily available in routine clinical practice. Second, implementation across diverse European healthcare systems highlighted variability in regulatory requirements, institutional approval processes, professional scopes of practice, reimbursement structures, and local infrastructure, which may influence how exercise services can be integrated into existing clinical pathways. Third, although the resource-stratified approach was designed to enhance transferability, practical implementation remains dependent on local resources, institutional prioritization, and the availability of trained personnel. Accordingly, the framework should be understood as a transferable implementation structure that requires local adaptation rather than as a universally prescriptive model.

## 6 Conclusion

Drawing on experiences from the multicenter FORTEe trial, this consensus statement offers resource-adapted recommendations to support the gradual development and expansion of exercise programs in childhood cancer patients.

The recommendations emphasize clearly defined interprofessional roles, standardized staff training, structured medical clearance and adaptation criteria, and systematic monitoring of exercise-related events to ensure patient safety. They also highlight flexible models for delivering exercise programs that can adapt to varying levels of staff availability, exercise space, equipment, and regulatory frameworks. A stepwise approach is proposed to enable centers to initiate exercise therapy within existing constraints and progressively expand services across resource domains.

Overall, the FORTEe framework demonstrates a potential practice-derived and expert-informed approach to implementing a flexible, inclusive, and adaptable exercise program across diverse pediatric oncology care settings, guided by principles of safety, feasibility, and patient-centeredness, and equitable access to supportive care. By emphasizing scalable and resource-sensitive implementation pathways, the framework may help support broader access to exercise oncology services across healthcare systems with differing organizational and economic capacities.

Although derived from the FORTEe trial context, the recommendations are intended to support other pediatric oncology centers in structuring and scaling exercise services according to their local resources and organizational capacities, and healthcare contexts.

## Data Availability

The original contributions presented in the study are included in the article/**Supplementary Material**. Further inquiries can be directed to the corresponding author.
