# Supplementary material for: Implementing exercise interventions in pediatric oncology: an expert consensus framework from the FORTEe project
Source: Front Oncol. 2026 Jul 8;16:1893935. doi: 10.3389/fonc.2026.1893935 (PMC13389839; doi:10.3389/fonc.2026.1893935)
Supplement: Supplementary file 1 [file Table1.docx]

FORTEe Recommendations for Medical Clearance and Reasons for Adapting Exercise

The FORTEe consortium developed study-specific medical-clearance criteria informed by both a review of the existing literature and expert consensus across participating trial sites. These recommendations outline indications for exercise clearance before both exercise testing and training, as well as criteria for modifying or temporarily withholding exercise in response to clinical findings. Medical clearance should ideally be performed jointly by the treating physician and the exercise professional and is tailored to each child prior to every exercise session.

Detailed criteria are published separately (1), core criteria include:

# Hematology:

- Children with platelet counts below 10,000/µL should refrain from exercise.
- For platelet counts below 30,000/µL, exercise should be individually tailored.
- Exercise should be individually adjusted if hemoglobin level is below 8 g/dL, as well as for patients with symptomatic anemia (e.g. dizziness, dyspnoea).

# Infection/Fever:

- Exercise is not recommended in the presence of fever above 38 °C or a severe or systemic infection.
- Mild, afebrile infections may allow for modified or adapted exercise.

# Bone tumors and risk of fractures:

- In patients with bone tumors or metastases, the affected region should not be loaded and treated with special care (in consultation with the treating physician).

# Central nervous system tumors:

- Patients with central nervous system tumors are at increased risk of falls and injury; exercise sessions should therefore be individually adapted considering paresis/ataxia, cognitive impairment, and seizure risk.

# Procedural Risk:

- Particular caution is required following biopsy or lumbar puncture and after surgical interventions

# Cardiovascular and respiratory disorders:

- Patients with cardiovascular disorders are at higher risk for lethal complication, especially acute, decompensated cardiac or respiratory conditions/ insufficiency should not exercise.
- Stable chronic conditions may permit exercise using adapted protocols.

# Treatment-related considerations:

- Chemotherapy: If possible, exercise sessions scheduled during ongoing/running intravenous chemotherapy should be avoided (e.g. due to the risk of dislocation of the central venous catheter), particular caution is during treatment with cardiotoxic agents.
- Radiotherapy: Exercise should be avoided during total body irradiation and mediastinal or cardiac-directed irradiation (up to 72 hours after the end of irradiation).

These clearance criteria ensure that each session is conducted within a framework of clinical safety while enabling participation whenever medically feasible.

# References

1. Neu MA, Dreismickenbecker E, Lanfranconi F, Stossel S, Balduzzi A, Wright P, et al. Get strong to fight childhood cancer - an exercise intervention for children and adolescents undergoing anti-cancer treatment (FORTEe): Rationale and design of a randomized controlled exercise trial. BMC Cancer. 2025;25(1):1275.
