# Supplementary material for: Implementing exercise interventions in pediatric oncology: an expert consensus framework from the FORTEe project
Source: Front Oncol. 2026 Jul 8;16:1893935. doi: 10.3389/fonc.2026.1893935 (PMC13389839; doi:10.3389/fonc.2026.1893935)
Supplement: Supplementary file 2 [file Table2.docx]

Documentation of Serious Exercise Related Complications (SERCs)

The reporting of (Serious) Exercise-Related health Complications (SERCs) in the FORTEe trial is structured based on the Common Terminology Criteria for Adverse Events (CTCAE) (1), utilizing its established code groups and specific codes to ensure standardized and consistent reporting of adverse events (2).

# SERC information

| SERC Number | ____________ | | |
| --- | --- | --- | --- |
|  | | | |
| SERC code group and  SERC code  *Please tick the SERC code group and specify the SERC code.* |  | Cardiac disorder (CD) | |
|  |  |  | Chest pain - cardiac |
|  |  |  | Palpitations |
|  |  |  | Other cardiac disorder |
|  |  | General disorders and administration side conditions (GDSC) | |
|  |  |  | Pain |
|  |  |  | Non-cardiac chest pain |
|  |  |  | Malaise |
|  |  |  | Other general disorders |
|  |  |  | Fracture |
|  |  | Injuries and procedural complications (IPC) | |
|  |  |  | Bruising |
|  |  |  | Fall |
|  |  |  | Other injuries |
|  |  |  | Arthralgia |
|  |  | Musculoskeletal and connective tissue disorder (MTD) | |
|  |  |  | Back pain |
|  |  |  | Bone pain |
|  |  |  | Myalgia (muscular pain) |
|  |  |  | Generalized muscle weakness |
|  |  |  | Muscle cramp |
|  |  |  | Other musculoskeletal disorders |
|  |  |  | Dizziness |

# References

1. U.S. Department of Health and Human Services NIoH, National Cancer Institute. Common Terminology Criteria for Adverse Events (CTCAE) v5.0 [Web]. Bethesda, MD: National Institutes of Health; 2017 [updated November 27, 2017. 5.0]. Available from: <https://ctep.cancer.gov/protocoldevelopment/electronic_applications/ctc.htm>.

2. Neu MA, Dreismickenbecker E, Lanfranconi F, Stossel S, Balduzzi A, Wright P, et al. Get strong to fight childhood cancer - an exercise intervention for children and adolescents undergoing anti-cancer treatment (FORTEe): Rationale and design of a randomized controlled exercise trial. BMC Cancer. 2025;25(1):1275.
