# Supplementary material for: Implementing exercise interventions in pediatric oncology: an expert consensus framework from the FORTEe project
Source: Front Oncol. 2026 Jul 8;16:1893935. doi: 10.3389/fonc.2026.1893935 (PMC13389839; doi:10.3389/fonc.2026.1893935)
Supplement: Supplementary file 3 [file Table3.docx]

Practical recommendations for exercise program implementation according to resource level in pediatric oncology settings

Supplementary Material 3. Practical recommendations for exercise program implementation according to resource level in pediatric oncology settings.

|  | **Resource Level** | | |
| --- | --- | --- | --- |
|  | **Basic Setting** | **Intermediate Setting** | **Enhanced Setting** |
| **Recommendations on Exercise Space** | - Deliver exercise sessions in non-dedicated but accessible areas, such as available ward spaces, corridors, or multipurpose rooms. Practical examples include:   - bedside training in single or shared patient rooms   - ward spaces and corridors   - stairwells (e.g. for step training if permitted)   - use of playrooms outside peak hours   - temporarily available consultation rooms   - unused seminar rooms during defined time slots - Mobile equipment carts can facilitate flexible bedside delivery - Prioritize locations that are easily reachable and visible to increase participation and facilitate medical oversight. - Maintain flexibility to adapt delivery to clinical workflows. | - Use small or shared rooms suitable/ dedicated for exercise. Practical examples include:   - physiotherapy rooms shared with other disciplines   - multipurpose therapy rooms   - partitioned areas within rehabilitation spaces - Where possible, establish semi-dedicated time windows reserved for oncology patients - Coordinate scheduling to ensure continuity and minimize disruption. | - Establish dedicated exercise facilities integrated into pediatric oncology care. Practical examples include:   - in-department exercise room / pediatric oncology gym   - formal collaboration with a hospital-based rehabilitation gym - Spaces should accommodate resistance training, endurance training (e.g. ergometer use), and free floor space for balance training, ball games, and playful activities - Design spaces to support individual and group-based training formats. - Ensure regular availability, clear visibility within the clinical environment, and reliable access for both inpatients and outpatients. - Proximity to treatment units will facilitate integration into routine care. |
| **Recommendations on Equipment** | - Use low-cost, portable, and disinfectable materials. Practical examples include:   - everyday objects such as filled water bottles or towels   - balloons   - resistance bands of varying strengths   - dumbbells   - ankle weights - Emphasize bodyweight-based strength exercises, sit-to-stand training, and playful movement tasks requiring minimal equipment. - Prioritize versatility and safety over specialization. | - Provide a basic but structured set of exercise equipment covering key training modalities. Practical examples include:   - therapy / medicine balls   - balance board   - step boards   - bedside cycle ergometers or pedal trainers   - dumbbell sets (adjustable dumbbells, higher loads)/ kettlebells   - semi-fixed multi-gym stations - Equipment should allow progression and individualization of exercise prescriptions, even if not all modalities can be optimally addressed. | - Ensure comprehensive professional equipment. Practical examples include:   - age- and size adapted cycle ergometers   - treadmills   - fully adjustable resistance machines for major muscle groups   - functional testing equipment   - interactive technologies, such as exergaming systems or virtual training platforms - Equipment should support precise load prescription, and long term progressive training across treatment and survivorship phases. |
